# Does Cancer Biology Rely on Parrondo’s Principles?

**DOI:** 10.3390/cancers13092197

**Published:** 2021-05-03

**Authors:** Jean-Pascal Capp, Aurora M Nedelcu, Antoine M Dujon, Benjamin Roche, Francesco Catania, Beata Ujvari, Catherine Alix-Panabières, Frédéric Thomas

**Affiliations:** 1 Toulouse Biotechnology Institute, University of Toulouse, INSA, CNRS, INRAE, 31400 Toulouse, France; 2 Department of Biology, University of New Brunswick, P.O. Box 4400, Fredericton, NB E3B 5A3, Canada; anedelcu@unb.ca; 3 Centre for Integrative Ecology, School of Life and Environmental Sciences, Deakin University, Deakin, VIC 3216, Australia; a.dujon@deakin.edu.au (A.M.D.); beata.ujvari@deakin.edu.au (B.U.); 4 CREEC/CANECEV, MIVEGEC (CREES), Centre de Recherches Ecologiques et Evolutives sur le Cancer, University of Montpellier, CNRS, IRD, 34000 Montpellier, France; benjamin.roche@ird.fr (B.R.); c-panabieres@chu-montpellier.fr (C.A.-P.); 5 Institute for Evolution and Biodiversity, University of Münster, Hüfferstrasse 1, 48149 Münster, Germany; fcata_01@uni-muenster.de; 6 Laboratory of Rare Human Circulating Cells (LCCRH), University Medical Centre of Montpellier, 34093 Montpellier, France

**Keywords:** cancer, dormancy, metastasis, Parrondo’s paradox, therapy

## Abstract

**Simple Summary:**

Parrondo’s paradox, whereby losing strategies or deleterious effects can combine to provide a winning outcome, has been increasingly applied by biologists to explain complex adaptations in many living systems. Here, we suggest that considering this paradox in oncology, particularly in relation to the phenotypic diversity of malignant cells, could also be a promising approach to understand several puzzling aspects of cancer biology. For example, the high genetic and epigenetic instability of cancer cells, their metastatic behavior and their capacity to enter dormancy could be explained by Parrondo’s theory. We also discuss the relevance of Parrondo’s paradox in a therapeutical framework using different examples. This work provides a compelling argument that the traditional separation between medicine and other disciplines remains a fundamental limitation that needs to be overcome if complex processes, such as oncogenesis, are to be completely understood.

**Abstract:**

Many aspects of cancer biology remain puzzling, including the proliferative and survival success of malignant cells in spite of their high genetic and epigenetic instability as well as their ability to express migrating phenotypes and/or enter dormancy despite possible fitness loss. Understanding the potential adaptive value of these phenotypic traits is confounded by the fact that, when considered separately, they seem to be rather detrimental at the cell level, at least in the short term. Here, we argue that cancer’s biology and success could frequently be governed by processes underlying Parrondo’s paradox, whereby combinations of intrinsically losing strategies may result in winning outcomes. Oncogenic selection would favor Parrondo’s dynamics because, given the environmental adversity in which malignant cells emerge and evolve, alternating between various less optimal strategies would represent the sole viable option to counteract the changing and deleterious environments cells are exposed to during tumorigenesis. We suggest that malignant processes could be viewed through this lens, and we discuss how Parrondo’s principles are also important when designing therapies against cancer.

## 1. Introduction

One of the most intriguing features of cancer cell populations is their high levels of stochasticity and plasticity states, especially in advanced cancers. It is increasingly evident that the associated non-genetic intratumoral heterogeneity (ITH) poses a significant challenge to cancer prognosis and treatment. For instance, phenotypic plasticity [1] and the interplay between genetic and non-genetic phenomena [2,3] have been recognized as very important factors in the emergence of resistant cells. It is then essential to fully understand both the proximate and the ultimate causes for the observed increased cellular stochasticity in cancer.

Many studies have provided evidence that cancer evolution and tumor dynamics are characterized by a progressive increase in epigenetic and gene expression diversity [4,5,6] (see below). In fact, high levels of non-genetic ITH coupled with plasticity might be general features of solid and hematological cancers. This global increase in diversity indicates that the contribution of cells with high plasticity and stem-like states increases during progression, and that their progeny harbor more diverse fates, with less defined gene expression patterns. Such a scenario can also easily explain the progressive dedifferentiation associated with increased aggressiveness that is usually observed in advanced cancers. 

The emerging question is why do cancer cell lineages experience such a global increase in stochasticity and transient fluctuations between infrequent expression patterns. In spite of detailed molecular studies providing proximate explanations for the observed increased epigenetic and gene expression diversity [7], ultimate causes and evolutionary explanations are still obscure. High levels of phenotypic heterogeneity associated with stochastic gene expression (even among genetically identical individuals) are also known in microbial populations [8]. Such heterogeneity allows populations to survive in fluctuating environments and promotes interactions among distinct phenotypic subpopulations leading to cell specialization (e.g., [8]). We have previously discussed the parallel between cancer cells and microbial populations from the point of view of cellular stochasticity (related to gene expression variability) and the way such stochasticity can be exploited to produce subpopulations better adapted to a given environment [9]. Specifically, we proposed that oncogenic processes rely on the initial increase in cellular stochasticity associated with cell de-differentiation, followed by the specialization of some cancer sub-populations while maintaining a less specialized lineage (cancer stem cells) with high stochasticity levels [9]. This can explain the co-existence of less specialized cells with higher stochasticity at the epigenetic and transcriptional levels able to diversify into many phenotypes, and more specialized cells with more stable epigenetic and transcriptional profiles that maximize exploitation of available resources in the surrounding environment. In this scenario, such unstable cells would be free to explore all the possible combinations of phenotypes thanks to their high plasticity. 

**Figure 1:**
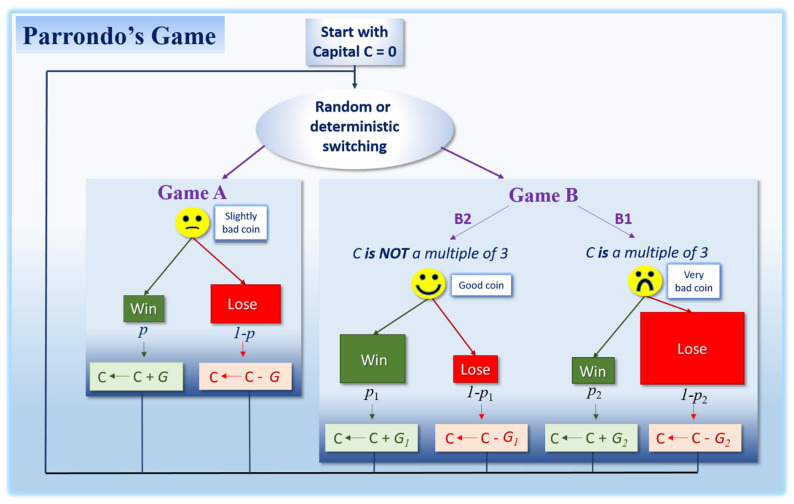
Parrondo’s original game, involving two games, A and B (adapted from [32]). See Box 1 for details.

Here, we go a step further by arguing that the observed increased cellular stochasticity and the presence of high plasticity states in advanced cancers could reflect the so-called “Parrondo’s paradox”, in which combinations of intrinsically losing strategies may result in wining outcomes (Figure 1, Box 1 and Box 2). While high plasticity and transient/fluctuating levels of gene expression could appear detrimental for growth, such traits (hereinafter also referred to as strategies) might be favorable in a highly disrupted and rapidly changing microenvironment, because they can prompt phenotypic diversification and thus enhance the tumor’s chance of success. In fact, maintaining a combination of highly plastic cells with transient gene expression and a set of more stable differentiated cells could constitute the only evolutionary strategy able to withstand the changing and deleterious environments that characterize advanced tumorigenesis. Gene expression noise and phenotypic diversification strategies have also been considered survival strategies for microbial populations in stressful environments [10]. We also argue that important characteristics of malignant cells, like their capacity to become dormant or to leave the primary tumor and migrate to other locations in the body, could be strategies that can be understood in the light of Parrondo’s paradox. 

## 2. The Origins of the Increased Epigenetic and Transcriptional Heterogeneity in Cancer

The increased epigenetic and transcriptional diversity that characterizes cancer progression could reflect an increased number of discrete cancer states among which cells can randomly fluctuate, resulting in increased phenotypic plasticity. The random fluctuations between distinct phenotypic states in cancer cell populations observed a decade ago argue for such a phenomenon [11]. The most usual hypothesis to explain this dynamic heterogeneity in cancer is based on the notion of cancer attractor state [12,13]. Indeed, cancer cells would explore parts of the global regulatory network that are not accessible to normal cells thanks to a reconfiguration of the epigenetic landscape (as defined by Conrad Waddington [14,15]) with the appearance of new valleys that would correspond to cancer attractors. Together with disruptive factors such as the abnormal microenvironment and the increased stochastic gene expression that would help cells to switch between attractor states, this cancer landscape would allow cells to experience gene expression patterns that are not observed in normal tissues. 

The increased epigenetic and transcriptional heterogeneity could also be, at least in part, the result of a global loss of chromatin coordination that is translated into single-cell phenotypic instability. The mutually exclusive activating and repressing histone modifications that co-map in single chronic lymphocytic leukemia cells [6] indicate that cancer cells lose at the epigenomic level the defined hierarchy of normal tissue, and can be characterized by co-occurrence of normally exclusive phenotypic markers. Previous studies already suggested that cancer cells revert to a ‘pseudo-primitive’ epigenetic status that combines features of embryonic stem cells and of different developing lineages [16], and that stochasticity of gene expression appears to be increased in cancer cells at higher levels than in normal stem cells because of a less organized and less stable chromatin structure [17]. Epigenetic regulators such as such as KDM5 family members have a key role in the generation of higher transcriptomic heterogeneity [7]. 

As recently shown in lung adenocarcinoma [4], cancer cells are characterized by a continuum of epigenetic states representing loss of cellular identities rather than by discrete and distinct states. This is consistent with previous data revealing that cancer should be conceived as a continuum of heterogeneous phenotype states because gradients of marker expression are observed rather than distinct subpopulations [18]. More generally, instead of being organized into well-categorized and discrete mature cell types carrying out specified functions as in normal tissues, cancer cells harbour increased plasticity and are distributed across a dynamic continuum of states from normal-like states with skewed differentiation to abnormal states [19]. 

However, some subpopulations are more plastic than others as recently shown in lung cancers [5]. In this case, it is proposed that highly plastic cells can give rise to more diverse fates, and are responsible for the emergence and maintenance of cellular heterogeneity. Thus, only certain subpopulations would really correspond to a state of high plasticity/instability that could be assimilated to a stem-like state with aggressive features, including robust potential for differentiation and proliferation as well as drug resistance, while the other subpopulations would harbor more discrete and stable states. Nevertheless, the highly plastic state does not overlap with the classical normal and cancer stem cell gene expression signatures [5], suggesting that high plasticity does not necessarily imply expression of stemness markers as classically defined. 

Epigenetic and transcriptional diversity could originate from cellular reprogramming that results in cells with stemness or plasticity, through pathological cell reprogramming processes involving abnormal stem cell signal activation and suppressor gene inactivation [20]. Especially, epithelial-to-mesenchymal transition (EMT) is expected to be a source of tumor heterogeneity [21] and to contribute to stemness and cell plasticity [22,23]. EMT is a hallmark of many different carcinomas, known to be associated with the initiation of metastasis [22,23]. However, it was recently shown that in breast tumors EMT is an inherent feature of most clones (which were found to harbor a major population of epithelial cells and a smaller and variable subpopulation of mesenchymal cells) [24], suggesting that the differentiation state of tumor cells is inherently unstable or plastic. Finally, since stochastic gene expression is normally controlled by cell-cell interactions [25], tissue disruption could produce a global destabilization of gene expression resulting in increased cellular stochasticity, and especially in high epigenetic and transcriptional diversity [26,27,28].

## 3. Parrondo’s Paradox in Cancer?

Based on the findings reviewed above, can Parrondo’s logics—that is, ‘losing/chaos + losing/chaos = winning/order’ underlie the persistence and/or progression of malignant tumors? Starting from the observation of a global increase in epigenetic and transcriptional diversity in cancer, the first cancer trait that should be explored through this angle is cellular instability/stochasticity (unstable and variable epigenetic landscape and gene expression pattern).

### 3.1. Are Both Stability and Instability Losing Strategies for Malignant Cells?

Building on Parrondo’s framework, we posit that, by itself, adopting a stable cellular state is a losing strategy because it can result in the death of the malignant cells. However, the association of this strategy with an alternation of transient states (which could be losing strategies on their own) can provide a winning outcome (e.g., by allowing persistence and/or proliferation in a changing environment). Is there evidence to support this possibility?

First indication about a possible involvement of Parrondo’s paradox in cancer comes from studies analyzing the role of gene expression variability in drug response. Specifically, it was shown that rare and transient gene expression patterns, which results from the inherent instability of cancer cells, fortuitously confer resistance to chemotherapeutic drugs [29]. In this pioneering work, the authors showed that the rare and transient transcription of a number of resistance markers at high levels in a very small percentage of single melanoma cells is at the origin of non-genetic resistance. Thus, a strategy consisting in increasing cellular stochasticity probably would allow a myriad of subpopulations that transiently harbor rare combinations of expression levels, ultimately leading to survival of a few resistant cells in a highly selective environment. More recently, the same authors found that groups of genes co-fluctuate in “coordinated rare-cell expression programs” that are heritable for several generations but ultimately transient [30]. One can generalize this observation by postulating that the stochastic appearance of a myriad of transient rare subpopulations expressing distinct gene expression patterns could be a general feature of dynamically fluctuating cancer cell populations.

Hinohara et al. also found a prominent role of gene expression variability in the emergence of resistant cells [7]. When the activity of members of the KDM5 demethylase family is inhibited so as to diminish gene expression stochasticity and reduce transcriptomic and phenotypic heterogeneity among estrogen receptor-positive breast cancers, resistance to endocrine therapies is reduced because fewer cells acquire resistance [7]. Thus, there is a clear relationship between the level of cellular stochasticity and the acquisition of drug resistance.

To extend these observations, we propose that this high stochasticity is also globally required for survival in the highly selective conditions found in advanced tumors because low or no variability would make cancer cells unable to deal with the changing disrupted environments that themselves and surrounding cells continuously modify. Cancer cell survival might rely, at least in part, on transient non-genetic changes that, although unfavorable in the current environment, may favor an adaptive response to future conditions (with some cells harboring the ‘right’ combination of expressed genes at every moment in every place), in line with the dynamic nature of the tumor microenvironment. 

Nevertheless, high instability by itself is likely a losing strategy. Indeed, recent single-cell analyses at the epigenomic level revealed no evidence that only highly unstable cells are present in advanced cancers; rather, cells with various levels of plasticity at various stages of tumor progression were found to co-exist [4]. These observations suggest that the co-existence of highly unstable and more stable subpopulations [5,9] should still be required for tumor maintenance and/or progression, even in advanced cancers. Furthermore, there is constant switch between the two states, as cells enriched for stem-like properties (i.e., unstable) known as tumor-initiating cells, can generate non-tumor-initiating cells, and the opposite [11,31], suggesting that the ability to change states (and the ratio between subpopulations expressing different states) is a necessary evolutionary strategy for tumor growth. 

**Figure 2:**
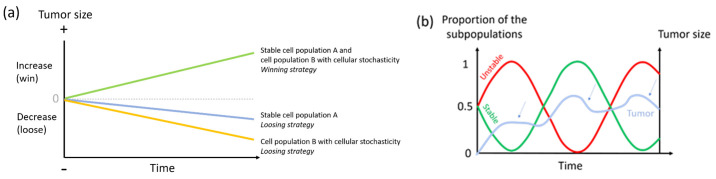
Hypothetical dynamics illustrating that (**a**) the co-existence of relatively stable cell populations and cell populations exhibiting stochasticity rely on the Parrondo’s paradox (adapted from [34]), (**b**) the co-existence of a relatively stable cell subpopulation (game A) and a cell subpopulation exhibiting stochasticity (game B) is necessary for tumoral growth (i.e., relying on Parrondo’s paradox). Tumor growth is impaired when the size of one subpopulation exceeds (or goes below) an optimal threshold of representation. The slope of the decrease depends on which unique strategy is overrepresented (small arrows). Also, the optimal ratio between the two strategies may vary during tumorigenesis, explaining the different slopes for the same ratios at different times.

Mathematically speaking, for Parrondo’s paradox to be at work, both a stable loosing strategy (Game A in Figure 1, see Box 1 for explanations) and a more stochastic loosing strategy (Game B in Figure 1, see Box 1 for explanations) are required, so that a ratchet effect producing the winning outcome can occur (it does not work without the ratchet). Thus, both a relatively stable cell population and a cell population exhibiting stochasticity are necessary for the Parrondo’s paradox to explain a tumor’s success (Figure 2a). Since the benefits from cell heterogeneity would depend on population size, the relative proportions between these stable and unstable subpopulations would vary depending on cancer stage, with the most advantageous ratio changing over time (Figure 2b). For instance, relatively more cells exhibiting elevated levels of variability might be needed at later stages to cope with an increasingly unpredictable and unstable micro-environment.

Box 1 **Parrondo’s paradox.**

Can the combination of two individually-losing strategies yield a winning outcome? Since the pioneering work of Harmer et al. [32,33], it has been accepted that this counterintuitive phenomenon, called “Parrondo’s paradox”, exists in a large variety of contexts [34,35]. A classical way to introduce Parrondo’s paradox considers a pair of losing games whose alternation provides a player with a winning outcome (Figure 1). Briefly, the basic principle is as follow: a player has some capital, which is increased by one unit when he/she wins, and decreased by one when he/she loses. In Game A, the player always loses in the long term because the game relies on a biased coin that slightly increases the player’s probability to lose each time the game is played (i.e., the loosing probability 1-p is slightly above 0.5). Game B is slightly more complex since there are two options depending on the player’s capital value: (i) if the capital is divisible by an integer (let’s say 3), the player uses Game B1 relying on a biased coin which is strongly unfavorable (9/10 chance to lose); (ii) if the capital is not divisible by 3, the player uses Game B2 relying on a biased coin that this time is favorable (3/4 chance to win). Despite this latter beneficial option B2, it can be demonstrated with discrete-time Markov analysis that Game B remains also on average a losing game [33,36,37]. Nevertheless, provided the unfavorable biasing parameter in Game A remains small, computer simulations (Brownian ratchet and discrete-time Markov chain) show that a player alternating the two losing games in a random or a deterministic manner will on average yield a winning game [33,36,37]. As it can be intuitively perceived, the construction of game B is a critical factor for paradoxical phenomena to emerge in Parrondo’s games (see [38]).

Parrondo’s principle has generated significant multidisciplinary interest in the literature, becoming paradigmatic for all situations (including in many biological contexts, see text) where losing strategies or deleterious effects can combine to provide a winning outcome (e.g., [39,40,41]). Interestingly, by exploiting Parrondo’s rule of alternating strategies, it can also be demonstrated that the periodic mixing of two chaotic dynamics can, in certain circumstances, result in ordered dynamics, illustrating a different Parrondian paradoxical phenomenon: “chaos + chaos = order” [42,43,44,45]. Although many aspects of cancer biology, like ITH and cellular stochasticity/instability are intriguing and somewhat counterintuitive, until now little attention has been devoted to exploring the extent to which cancer’s success could, at least partially, rely on Parrondo’s principles. Nevertheless, some applications of the Parrondo’s game to cancer in a theoretical modeling and chaos control framework can be found in the literature [46,47], especially showing that switching control parameters can make a previously chaotic tumor growth trajectory nonchaotic, and inversely [47].

Box 2 **Parrondo’s effects in biology.**

Although Parrondo’s paradox has initially received a lot of attention from mathematicians and physicists, it is increasingly recognized that the genetic, ecological and evolutionary dynamics of numerous living systems (from genes to populations) can also, to some extent, be influenced by Parrondo’s effects. Few examples are presented below (but see [48] for a recent review).

In genetics, Reed [49] used Parrondo’s logic to explain how, in a sexually reproducing species, an autosomal allele that is, on average, deleterious for each sex (compared to an alternative allele) can nevertheless increase in frequency, persist in the population, and even continue to fixation. This situation is possible when the detrimental autosomal allele enhances fitness in combination with an allosomal allele in females only (i.e., a positive epistatic interaction coupled with a sexually antagonistic selection). This optimal context, equivalent to Game B2 in Figure 1 (see also Box 1), inevitably occurs in alternation because of genetic inheritance processes arising in sexual reproduction, allowing individually-losing strategies to be temporally intercalated to yield winning outcomes.

In bacteria, random phase variation (RPV; that is, unpredictable transitions between alternative states) is a strategy often favored in environments that are rapidly changing in time or space, even if some of the resulting phenotypes are likely to be at any time maladapted to the current environment. This suboptimal strategy implies that bacteria evolve imperfect ways to detect environmental transitions, with selection actually favoring individuals with sensors of lower accuracy and/or with enhanced signal transduction delays. Because RPV populations display on average a reduced growth rate variance, they may also become in the long term vulnerable to extinction when all extrinsic factor variations are considered. Interestingly, Wolf et al. [50] demonstrated with a game-theory model that a mixed stochastic/deterministic strategy can emerge as an evolutionarily stable strategy. Thus, random alternations between losing strategies likely to produce bacteria with the wrong phase variation or sequence of variations, can result in a winning outcome, especially when the regimes of bacterial switching rates match the rate of environmental instability (see also [51,52,53]).

Parrondo’s effects have also been proposed as a possible explanation for the enigma of populations able to persist in environments exclusively composed of sink habitats. Jansen and Yoshimura [54] showed that such a persistence becomes possible when habitats’ quality fluctuates through time and offspring produced can disperse between habitats. In a similar vein, Cheong et al. [55,56,57] showed that a mix of two losing lifestyles, called nomads and colonists, can result in a winning outcome. In their example, nomads are independent and neither compete nor cooperate, and rely on relatively low levels of resources. Being a nomad is a losing strategy because in the long term, individuals do not reproduce at a rate that compensates losses in death associated with this life-style. Being a colonist is also a losing strategy because even if individuals in this state have access to more resources and interact (cooperation but also competition), sooner or later they deplete their habitat and cannot persist. The switching between these two losing strategies not only ensures that population extinction is avoided, it can even facilitate proliferation if, for instance, colonists (i) strategically switch to a nomadic lifestyle when the over-exploited resources of their habitat become scarce, and (ii) switch back to a colonist lifestyle once novel resources are found (e.g., a novel habitat or once their habitat is replete). The cellular slime mold *Dictyostelium discoideum* may provide a possible example. These amoebae have a life cycle including a single-cellular stage and a multicellular one, when individual amoebae aggregate. Selection for aggregation occurs only in environments where food is slow to replenish, otherwise unicellularity is most of the time favored. While each strategy is intrinsically a losing one in the long term, alternating the two lifestyles depending on the food availability is a winning one [58].

Since research on Parrondo’s paradox has been extended into ecology and evolutionary biology, it is increasingly apparent that this phenomenon is an important, if not a major process to consider when attempting to explain numerous general features of life, including adaptive as well as apparently maladaptive traits. For instance, its significance is illustrated by the recent provocative suggestion that the evolution of alternating unicellular and multicellular life history stages that enabled the success of multicellular lineages involved Parrondo’s dynamics [59,60]. Surprisingly, the possibility that aspects of cancer’s biology and success could, at least partially, rely on Parrondo’s dynamics has received little attention.

### 3.2. Why Should Cell Populations with High Instability/Stochasticity Be Needed for Long Term Survival and Proliferation?

To date, all theoretical and empirical examples of Parrondo’s paradox in biology have considered situations involving the mix of only a few losing strategies, usually two. Given the large diversity of cellular phenotypes expressed through time by unstable and stochastic cells, one might question if these phenotypes are indeed manifestations of different losing strategies, and why Parrondo’s dynamics in malignant cells needs so many alternative strategies.

A first possible explanation comes from the fact that a major difference between malignant cells and other living forms is that the former do not possess adaptations finely-tuned by selection over millions of years. Except for transmissible cancers, each cancer must ‘reinvent the wheel’ as their evolutionary products die with the host. In the same way, the bet-hedging of malignant cells may appear rudimentary/primitive compared to, for example, that of desert plants for which only the seed germination timing is variable. The fine tuning of the potentially adaptive combination of strategies underlying the Parrondo’s logic in malignant cells is not possible in such a short time. Differently put, the genesis of a biological substrate underlying a Parrondo’s logic in malignant cells could lack sophisticated calibration. If this hypothesis is correct, then Parrondo’s dynamics in transmissible cancers, which have the opportunity to evolve over longer period of time (beyond the lifespan of their hosts), should rely on fewer options, resulting in a reduced cellular instability/stochasticity compared to normal cancers. Similarly, examples of Parrondo’s dynamics in micro-organisms like bacteria should rely on fewer, better adjusted, losing strategies [50].

A second possible explanation for the elevated number of “losing/suboptimal strategies” in cancer cells is that the environmental conditions experienced by malignant cells are so unstable, diversified and adverse, that any viable Parrondo’s dynamics could only rely on a myriad of strategies to be sustainable. From an initially normal cell’s perspective, the tumor environment is indeed characterized by a high level of adversity and instability [61,62]. These unfavorable ecological conditions may originate from the host (microenvironment, immune system), but also from the malignant cells themselves since their activities and proliferation largely contribute to altering, in a non-predictable manner, numerous variables in the tumor environment. Because malignant cells typically die with their hosts, these unprecedented ecological conditions result each time in a novel habitat for which no specific (cross-generational) adaptation could have evolved. In this context, a cellular instability/stochasticity exploited within a Parrondo’s logic may be selected as a viable option.

Another non-mutually exclusive explanation could be a run-away process, initiated by external ecological factors and then maintained and amplified by internal cell factors. Cellular instability/stochasticity would not only permit malignant cells to cope with unprecedented environmental conditions (see above), but it would also impair the cells’ normal functioning by introducing genetic/epigenetic abnormalities. While in response to such abnormalities healthy cells usually activate apoptosis, oncogenic selection in malignant cells is likely to favor increased cellular instability/stochasticity; this diversification will favor many possible survival strategies. However, an increase of cellular instability/stochasticity is likely to introduce additional internal cell impairments that will subsequently reinforce the selection for an exacerbated Parrondo’s compensatory response via a novel enhancement of the cellular instability/stochasticity etc. In this runaway scenario, it is expected that the levels of cellular impairment and instability/stochasticity are positively correlated and should increase during tumorigenesis, until a level for which cell instability/stochasticity probably becomes insufficient to compensate, via Parrondo’s effects, all cellular dysfunctions. 

### 3.3. Dormancy: A Losing Strategy in Parrondo’s Dynamics?

Dormancy and quiescence are frequently observed in malignant cells [63]. Although advantageous in adverse conditions, dormancy and quiescence may be considered a losing strategy because cells that would permanently switch toward this lifestyle are exposed to the risk of dying without producing offspring. A switch toward these states of suspended growth seems even more surprising when it occurs in environmental conditions that are a priori not adverse for malignant proliferation [64,65]. Alternating dormancy with other malignant lifestyles could however be interpreted as a mixed strategy shaped by Parrondo’s dynamics. Malignant cells that are highly proliferative have the advantage of reproducing rapidly, but experience a higher mortality rate if conditions become unfavorable, e.g., because of changes in the microenvironment and of therapies (i.e., the major focus of therapy development remains on directly targeting viability or proliferation of tumor cells). Conversely, dormant/quiescent malignant cells exhibit a lower reproduction rate, but they benefit from increased survival under adverse conditions, as illustrated by the relapse they cause years, even decades, later. These two phenotypes (i.e., high proliferation and suspended growth) correspond to fast and slow life-history strategies, respectively. In unstable environments, organisms frequently adjust by adopting different phenotypes in response to different external conditions [66]. However, when reliable cues for predicting environmental changes are lacking, and/or when populations did not evolve the capacity to exploit them (as expected for malignant cells that have at best few years of evolution [67]), individual organisms are often constrained to develop a strategy based on a stochastic switching between different phenotypes/stages (i.e., a bet-hedging strategy [68]). Because malignant cells can only respond to direct selection forces without the possibility to anticipate future conditions and dynamics of the ecosystem, dormancy/quiescence may be a part of a generalized bet-hedging strategy. In summary, stochastic alternation between strategies through the entry in and exit from dormancy/quiescence does not maximize fitness within a generation, but it reduces fitness variance and hence maximizes tumor’s success under environmental unpredictability in the long term. This ultimately represents a winning outcome based on the combination of two losing strategies—that is, a case of the Parrondo’s paradox.

### 3.4. Is the Metastatic Behavior a Strategy within Parrondo’s Paradox?

Metastasis remains the leading cause of mortality for cancer patients [69]. However, the proximate and ultimate causes of this phenomenon are not completely understood [70]. As proposed by many evolutionary ecologists and cancer biologists, metastasis could be considered through the lens of biological dispersal [71]. In the light of the previous literature on the colonist/nomad game (see Box 2), it is useful to explore the metastatic process within the framework of Parrondo’s paradox. The fact that less than 0.1% of cells disseminating from the primary tumor form metastases (a phenomenon referred to as ‘metastatic inefficiency’ [72]) suggests that leaving the primary tumor to disperse is a costly, extremely frequent, losing strategy [73,74,75]. Can the non-metastatic behavior also be viewed as a losing strategy, i.e., equivalent to a colonist losing strategy? This possibility is consistent with events that unfold in certain parts of solid tumors, especially as resources and/or space inevitably become limiting. Angiogenesis is critical for tumor survival, and once solid tumors reach a substantial size, vascularization becomes irregular in the core regions yielding tumor necrosis [76,77]. Within the Parrondo’s logic, there should be a positive correlation between the frequency of necrosis phenomena and the propensity of malignant cells to adopt a dispersing lifestyle. Accordingly, clinical observations clearly indicate that tumor necrosis of solid tumors is an accurate indication of metastatic tumors [78,79,80,81,82,83,84]. In the same context, a link has been established between acidification [85] or the lack of oxygen [86], and the propensity to metastasis, revealing that a deleterious environment for the colonists in the primary tumor can lead to another mainly inefficient and deadly strategy for malignant cells, with very rare successes. Thus, both loosing strategies could lead to a winning outcome when a few metastatic cells succeed in dispersing, invading new tissues and resume proliferation, because they have both the prerequisite malignant phenotypes and the available resource and space in the new environment.

## 4. Therapeutic Implications

Malignant cells can become resistant to many different types of drugs [87,88]. High and continuous doses of drugs typically allow resistant cancer cells to win twice. First, they are not killed; second, they are not outcompeted by sensitive cancer cells. Because of this combination of effects, treating cancers with such protocols seems to be a losing strategy because it ultimately results in fatal disseminated cancers. However, applying no treatment is often a losing strategy too because cancer progression is not prevented, yielding also to disseminated cancers that kill patients. Alternating these two strategies can produce a better option, as elegantly illustrated by the adaptive therapy approach [89]. Acquisition of chemo-resistance generally requires significant investment of resources, and because of the ‘cost’ of phenotypic resistance, cancer cells are subject to an evolutionary trade-off between resistance and proliferation. Adaptive therapy alternates treatment and treatment breaks in an adaptive fashion to enforce a stable tumor burden by permitting the persistence of a significant population of chemo-sensitive cells. In so doing, chemo-sensitive cells can compete with chemo-resistant subpopulations hence limiting their expansion. Instead of introducing treatment breaks, another strategy consists in providing fake drugs. Cells that are resistant to multiple drugs often have efflux pumps that remove the drugs. The cell pumps, however, require energy to run. Fake drugs proposed by Kam et al. [90], also called ‘ersatzdroges’, are non (or minimally) toxic substances that activate efflux pumps in resistant cells and cause them to expend energy, without actually giving these cells a survival benefit over non-resistant cells. Applying only these ersatzdroges is a losing strategy because it does not prevent the proliferation of cells that do not possess pumps and the tumor can continue to grow. However, alternating a fake drug with a real drug can keep the size of tumor constant, without resistance selection.

Inspired by the processes responsible for the extinction of species, Gatenby et al. [91] demonstrated that once an initial therapy reduced population size and diversity of a large tumor, several less aggressive therapies, each unable to eradicate large tumors, can successfully eliminate small and spatially fragmented malignancies, without selecting for resistance (e.g., neo-adjuvant chemotherapy). Again, while each category of treatment is unable to prevent cancer progression, alternating them in the order proposed above yields a relevant therapeutic option, illustrating the use of Parrondo’s paradox in a therapeutical framework. 

## 5. Concluding Remarks

Although tumor progression has stimulated a large number of clinical and theoretical studies, its underlying mechanisms still remain elusive. The extent to which oncogenic selection promotes malignant strategies relying on Parrondo’s paradox principles is a legitimate question, at least because the instability and the multidimensionality of cancer cells’ phenotypes do not systematically correspond to alterations that effectively contribute to increase their fitness at any time. Studies that focus on occasional snapshots of the tumor state cannot capture these Parrondo’s dynamics. Further work is also necessary to explore the extent to which malignant cell switching rates match optimally or not the rate of micro environmental instability (i.e., as for the random phase variation with bacteria discussed above). Another relevant direction would be to test the hypothesis that selection for Parrondo dynamics generates syndromes in malignant cells, that is the simultaneous alteration of multiple phenotypic traits, that could appear not optimal individually but that collectively produce a winning outcome. These syndromes would result from some major physiological disruptions reflecting the need to combine many losing strategies to acquire full malignancy. The complexity and the sub-optimality of malignant phenotypes could, thus, at least partially, result from few major physiological effects selected under Parrondo’s logic, followed by a cascade of phenotypic effects forming a syndrome. In conclusion, future exploration of the Parrondo’s paradox and its relevance to the phenotypic diversity of tumor cells could provide novel insight into the complexity of cancer dynamics and progression.

## Data Availability

Not applicable.
